# Health and Hell

**Published:** 1889-06

**Authors:** S. H. Preston


### HEALTH AND HELL.

BY S. H. PRESTON.

No. 2.

As already remarked, no man in normal health ever really believed in hell. Indeed, it may be maintained that no man not insane ever adopted the dreadful doctrine of damnation. Why, earth would have become an enormous madhouse ere this, had its habitants entertained the terrible thought of eternal torment. But the dogma has been too horrible for human credence or comprehension.

As a brief and blasphemous by-word, hell has been bandied about till folks have forgotten its frightful significance. And people professing belief in it as a fiery reality have only befooled their own brains, for they could form no idea of anything so inconceivably infernal, and hence, could not formulate it into their faith. During the dark old days of ignorance and fear people pretended to think there was such an awful place, because they were told they would be damned if they did not thus believe. And so the slaves of superstition, under duress of doctrinal despotism, assented to something that shocked their souls and staggered their understanding. But few had any belief in it, because no human being could believe in it till his liver and lights, stomach and bowels and bladder got into the terrible condition of John Calvin's.

And precisely, according to the progress made in the practice of medicine, by which many of the malignant maladies of mankind have been completely overcome, as the depopulating plagues of the Middle Ages, according to the introduction of anæsthetics and remedies for the alleviation of human suffering, has hell lost its horrors and its hold on Christian credulity.

The afflictions of our ancestors were acute, and their methods of medical treatment truly heroic. Their way of amputating a leg, by chopping it off with a big cleaver and cauterizing the stump in a cauldron of boiling pitch, might be called hellish in our day of chloroform and scientific surgery. And their hell *was* a hell—a sulphuric old scorcher—not such a sham and fireless affair as now goes by the name of hades. Its redhot highways and burning boulevards were macadamized with the souls of span-long babes. A good deal of the fuel in the old Presbyterian perdition was furnished by infants. According to Jonathan Edwards, "reprobate infants are vipers of vengeance, which Jehovah will hold over hell in the tongs of his wrath till they turn and spit venom in his face." And the old-time saints used to sing such sacred songs as this:

"There is a never-dying hell,
And never-dying pains,
Where children will with demons dwell
In darkness, fire and chains.
Have faith the same in endless shame,
For all the human race,
For hell is crammed with infants damned
Without a day of grace.

Hell-fire got considerably quenched by the time doctors let their fever patients take a drink of water. The blue blazes went out as the administration of blue mass was abandoned. The doctrines endorsed by Calvin were modified or discarded as doses of calomel diminished. Hydropathy turned a hose on hell that made quite a hissing for a time. And more water dripped into hell while homœpaths were diluting their drugs. And every new school of medicine, every advance in the art of healing, has knocked down the thermometer a degree in perdition, till already its climate has become quite comfortable for an unclad spirit. Hell has been revised into hades, and is now regarded merely as a heavenly half-way house or retreat just beyond the border—a kind of eternal Montreal.

Though John Calvin was a very unhealthy man and a good deal like the God of the Old Testament, he was not, as has been shown, the inventor of the infant damnation doctrine. The idea of hell was born in the brain of the sunburnt barbarian. He abode in some such blistered region as Arabia, and the "glorious god of day" appeared to him as a malignant monster, smiting him with its rays of wrath as it crawled slowly across the sky. Having discharged its stock of heat during the day, it descended into a burning abyss beneath the earth to fire up again for its next daily course. He shouted defiance at his dazzling foe and shot his rude darts at its scorching beams all in vain. Indigestion, caused by his uncooked food, gave him distressing dreams. Frightful fancies of the fiery furnace below racked his brain. He felt the breath of the viewless powers of the air, and stood appalled at the awful mystery of death. The barbarian brain is that of a child.

In the course of time, a class called priests arose, who made it their exclusive business to explore the unseen realms and unriddle their secrets. These priests flourished upon the fears of their superstitious fellows.

And from such gross and grotesque notions of nature and of her vague and varied powers as occurred to these first child-men of the race, were eventually elaborated all the religious systems of antiquity ; the crude and ceremonial faith of Egypt, a combination of the silly and sublime, the picturesque symbolism of Etruria, the magnificent materialism and adoration of Persia, the Median dualism, the impure worship of Mylita and Astarte, the cruel rites of Carthage, the Druidism of Gaul and Britain, and the complete cycle of religions gathered at last into the grand and complicated mythologies of Greece and Rome.

And then came the ages of books. And there was a venerable book that described the state of the damned, and spoke of the smoke of their torments ascending up forever and forever. This book became the beacon of the Christian world, because of the belief that God gave it. And John Calvin, afflicted with an inflexible faith as well as with inflamed stomach and stone in the bladder, literally believed every word of it. From this guide book of God were compiled the creeds of Christendom, and from them Calvin formulated those fearful "five points," that for three centuries have bristled like hell-hot spikes upon the panoply of Presbyterianism. Those "points" became firmly fixed, being rivetted by Bible bolts. Theologians and commentators died in despair trying to twist the stubborn texts into more merciful meanings. And there were just and humane men who would gladly have given their lives to discover some different interpretation. But turn these texts topsy-turvy or this way and that, there pricked those terrible "points." And the just men knew they were unjust and the humane men felt they were fiendish ; but there they were, God's own words, and "O, man, who art thou that repliest against God ?"

The reason we refer to Calvin so frequently in this connection, is because his name has come down to us associated with all that is execrable and cruel in the old hell-fire faith. The mere mention of him seems to bring up a blast of brimstone from the pits of perdition, where infants writhe in eternal torment. In thinking of hell one cannot help thinking of Calvin, and vise versa. But, fair play to his name, he did not write the book that revealed the hell, or claim any caveat for the Presbyterian creed. He devoutly believed the book and accepted the doctrines that damnify the creed. That was all. As he was, he could not help believing in hell. He was full of hell physically

—an impersonation of Tophet. There was hell in his feverish brain, his dyspeptic stomach, gouty feet, graveled bladder, diseased lungs and liver and kidneys. His health was bad, and hence, his hell was awful.

While a man is not responsible for his belief, that is, while he cannot believe as he wills, he is made either a better or a worse man thereby. He cannot reasonably be expected to be better than the being or book, the idol or ideal, that he worships. But even a sculptored idol, if it be the production of a Praxateles, may image its marble majesty and beauty through the windows of the eyes upon his soul, and bring him a better and more adorable ideal. Ancient Athenian mothers were enabled to breed a better race by constantly contemplating the statues of Apollo and Narcissus set up in their chambers. So that an image erected of golden thoughts to represent a grand and perfect man, a moral model set up like a mirror in the chamber of conscience, before which man may adorn and purify his soul, though, but a religious illusion, will not fail to ennoble his nature and lift his life to a loftier level of thought and action. But if the image built up in the brain to be honored by the heart be something base, or crude, or cruel, then will it dwarf and degrade him, and contaminate his character the more he contemplates it.

Simple credence of a creed or blind adoption of a dogma is a different matter. A belief that the world was made by one God, or three, or thirteen, will not necessarily make a man any better, but it may imbue him with bigotry against those who believe in a different number of Gods. And this is the evil of cast iron creeds and Procrustean theologic systems. They put belief before goodness, doctrine before duty. To attend a certain church, participate in prescribed ceremonies, and promptly pay the pew rent, is deemed of more importance than correct conduct and a moral life. Thus, it is frequently found that a godly man is far from being a good man; a pious, prayerful man is not always a pure and principled one; and it may happen that a heavenly-minded man may be a mean, hard-hearted and dishonest man. If guilty of some flagrant offence against a fellow being which frets his conscience, he reads his Bible and goes to church with more regularity, and increases his subscription for the pastor's salary. It is thus that artificial virtues are sometimes offset against actual vices.

Now, it may be replied that there live, to-day, really healthy people who believe in hell. Please point them out, and note if their apathy does not belie their professed belief—does not prove them to be only lip believers. Do they act as if they actually believed anybody was going to be burned in eternal fire? If they thought the house of even a hated neighbor was on fire, would they rest till they had rescued every one of the inmates, dog and cat and canary bird included? Would they be likely to leave the imperilled lives to be looked after by some sectarian insurance company, or stop to draw up a petition to the fire department. Why, the worst wretch who lives in this world would, were he convinced that there were babies burning in hell, try to put out its infernal fires with his tears. By the time a man does become a believer in hell his health breaks down or he becomes an inmate of an insane asylum.

The next number will comment on Calvin's part in the burning of Servetus—an act eminently exemplifying hell on earth.

